# Reply to comment by Normanno *et al* on ‘A comparison of three methods for detecting KRAS mutations in formalin-fixed colorectal cancer specimens’

**DOI:** 10.1038/bjc.2012.432

**Published:** 2012-09-25

**Authors:** D G de Castro, F Lopez-Rios

**Affiliations:** 1 Molecular Diagnostics Department, The Institute of Cancer Research and the Royal Marsden NHS Foundation Trust, 15 Cotswold Road, London SM2 5NG, UK; 2 Laboratorio de Dianas Terapeuticas, Centro Integral Oncologico Clara Campal, Hospital Universitario Madrid Norte Sanchinarro, C/Ona, 10, 28050 Madrid, Spain

**Sir,**

The letter by Normanno *et al* in reference to our manuscript recently published in the *British Journal of Cancer* raises very interesting issues that are pertinent to routine *KRAS* testing in molecular pathology practice and we would like to add our comments to help clarify such issues:

As Dr Normanno *et al* (2012) pointed out, the cobas *KRAS* mutation kit was highly reproducible between laboratories, irrespective of the levels of necrosis, lymphocyte or tumour percentage. This is normally expected from commercially available kits for *in vitro* diagnostic use, but it is perhaps enhanced here by the fact that the cobas kit is linked to specific instrument and analysis software providing automated genotyping calls and, therefore, reducing the subjectivity of the interpretation. We fully agree with the comment that in most laboratories macro-dissection is performed before Sanger sequencing and, therefore, the limit of sensitivity may not be such an issue. Nonetheless, with this in mind we designed the study a priori purposely avoiding macro-dissection for two reasons: first, we wanted to minimise manual handling of specimens to avoid any biased difference between the material used for the different methods, as well as potential human errors while macro-dissecting the tissue, and, therefore, comparing strictly the methods’ performance on the same starting material; second, in our clinical practice we receive samples where macro-dissection may not be possible because of scanty specimens being available (although this is less common in CRC) or due to a high lymphocyte infiltration with <30% tumour content, and, therefore we thought it would be more informative to perform the studies without macro-dissecting the tissue.

The main objective of our study was to perform a comparison study of three different methods for *KRAS* mutation detection in clinical diagnostic laboratories. Our manuscript is not intended to provide clinical guidelines on interpretation of the different mutations and we tried to make clear in the introduction and discussion sections that although there is emerging data about the role of codon 61 mutations in relation to anti-EGFR antibody therapies, there is a need for additional clinical studies to confirm the data before introduction in routine clinical practice. In this sense, the cobas *KRAS* mutation kit allows the detection of codon 61 mutations, and these can be reported distinctively from codon 12/13 mutations if required, enabling the pathologist to make informed decisions based on the available and emerging clinical evidence, as well as local practice or guidelines. Currently, the EMA labelling for cetuximab and vectibix state that these drugs are contra-indicated in patients with *KRAS* mutations without specifying which mutations should be tested for. We are aware that most of the evidence in clinical trials has included codons 12 and 13 only, and we believe it is important to expand our knowledge in the routine clinical setting as this can lead to more cost-effective therapies and improve outcomes for CRC patients. Nonetheless, we would like to stress that the information regarding codon 61 mutations in the context of clinical outcome needs to be extensively evaluated and its interpretation in routine clinical reports must be performed with great caution until further data is available.

We agree with Normanno *et al* that experienced molecular pathologists can interpret complex patterns of results with any given technology and this could lead in certain cases to a different diagnosis. Unfortunately, we have no data to infer that manual interpretation would have resulted in more accurate results in our study. Furthermore, we have no evidence in our study that if the cobas results would have been subjected to manual interpretation it could not have lead to a higher level of false positive and/ or false negative cases. In fact there was one false negative by the Therascreen test and another one by Sanger sequencing, in both instances after manual interpretation by experienced molecular pathologists. We believe that closed systems with automatic analysis and reporting algorithms represent a step forward in routine molecular pathology, as it has been the case in other pathology disciplines such as microbiology/virology, helping to ensure reliability and reproducibility even in centres with less experience in molecular methods.

We find this last issue of the inability of the cobas KRAS kit to distinguish between codon 12 and 13 mutations slightly contradictory to the second point raised by Normanno *et al* regarding codon 61 mutations. We agree with the authors that the cobas KRAS kit is not suitable for a research analysis of the impact of the different codon 12 and 13 mutations with regard to targeted therapy. As suggested by Peeters *et al* (2011) we believe that these studies need to be performed with control series in the context of randomised trials. In contrast, the information regarding codon 61 mutations can be currently omitted from clinical decision-making if required by local guidelines (i.e., considering a codon 61 mutations as ‘wild-type’ *KRAS* sample) and perform retrospective studies in large populations in relation to outcome in the future.

In conclusion, our comparison study does not aim to recommend a particular technology, as several technologies have been proven to be adequate for *KRAS* mutation testing in the clinical setting, depending on laboratory experience and expertise. We provide evidence on the performance of different methodologies from a technical point of view that we hope are informative enough for individual laboratories to decide on the most suitable testing strategy according to their local needs and clinical evidence.
